# Acquisition of the dorsal structures in chordate amphioxus

**DOI:** 10.1098/rsob.160062

**Published:** 2016-06-15

**Authors:** Arseniy R. Morov, Tharcisse Ukizintambara, Rushan M. Sabirov, Kinya Yasui

**Affiliations:** 1 Department of Biological Science, Graduate School of Science, Hiroshima University, 1-3-1, Kagamiyama, Higashi-Hiroshima, Hiroshima 739-8526, Japan; 2 Department of Zoology and General Biology, Institute of Fundamental Medicine and Biology, Kazan (Volga Region) Federal University, 18 Kremlyovskaya Street, Kazan 420008, Republic of Tatarstan, Russian Federation

**Keywords:** lancelet, chordate origin, deuterostomes, dorsoventral inversion, dorsal formation

## Abstract

Acquisition of dorsal structures, such as notochord and hollow nerve cord, is likely to have had a profound influence upon vertebrate evolution. Dorsal formation in chordate development thus has been intensively studied in vertebrates and ascidians. However, the present understanding does not explain how chordates acquired dorsal structures. Here we show that amphioxus retains a key clue to answer this question. In amphioxus embryos, maternal *nodal* mRNA distributes asymmetrically in accordance with the remodelling of the cortical cytoskeleton in the fertilized egg, and subsequently *lefty* is first expressed in a patch of blastomeres across the equator where *wnt8* is expressed circularly and which will become the margin of the blastopore. The *lefty* domain co-expresses zygotic *nodal* by the initial gastrula stage on the one side of the blastopore margin and induces the expression of *goosecoid*, *not-like, chordin* and *brachyury1* genes in this region, as in the oral ectoderm of sea urchin embryos, which provides a basis for the formation of the dorsal structures. The striking similarity in the gene regulations and their respective expression domains when comparing dorsal formation in amphioxus and the determination of the oral ectoderm in sea urchin embryos suggests that chordates derived from an ambulacrarian-type blastula with dorsoventral inversion.

## 1. Background

As human beings are derived from chordates, research on the chordate body plan has fascinated zoologists since the study by German zoologist Haeckel [1]. Among the features of the chordate body, dorsal structures such as the notochord and the hollow nerve cord are most conspicuous, and great efforts have been made to investigate the mechanisms underlying the development of the dorsal structures since the discovery of the amphibian organizer by Spemann & Mangold [2]. We currently understand that maternal mRNAs related to the formation of the morphogenetic centre (dorsal organizer) and associated intracellular structures, which are mostly localized at the vegetal pole during oogenesis, provide the foundation for vertebrate dorsal formation [3]. By sperm entry on the animal hemisphere that triggers the cortical reaction, finalizing meiosis and starting the mitotic cell cycle, and the relocation of maternal factors facilitated by microtubules [4–6], a fertilized egg is reorganized from its radial symmetry to future bilateral symmetry with anteroposterior, dorsoventral and left–right axes. Through this reorganization, the centre of gastrulation and the initial morphogenetic centre are separated eccentrically, and the latter is located on the one side of the future blastopore margin [7]. Ascidians also display ooplasmic dynamics more remarkably than anamniote vertebrates do, however they do not specify the dorsal fate but the posterior fate by this process [8,9]. Dorsal structures are induced by asymmetrical segregation of mRNAs during cleavage and Fgf signalling emanating from endodermal precursor cells [10,11].

Amphioxus, now regarded as the sister group of vertebrates + urochordates [12,13], shares a common chordate body plan with the latter group, collectively called Olfactores [14]. However, amphioxus eggs display some different behaviours from those of olfactoreans when fertilized. The amphioxus egg localizes some maternal mRNAs such as *nodal*, an important mRNA for the initial morphogenesis, in the animal hemisphere [15] and does not undergo a remarkable rearrangement of maternal factors, contrary to as in anamniote vertebrate and ascidian eggs [16]. Furthermore, nuclear localization of β-catenin, which is one of the earliest molecular markers for the dorsal determination in vertebrates and for endomesodermal differentiation in ascidians [17,18], occurs in every cell by the 64-cell stage [19,20], and thus cannot be used to predict the dorsal side. Even though amphioxus has these differences from the other chordate groups, it displays gene expression patterns comparable to those in vertebrates during gastrulation [21], which gives rise to a body pattern very similar to the vertebrate pattern.

Sea urchins, a member of Echinodermata, which is a sister clade of Chordata, are well characterized in their development [22,23]. As they develop penta-radial body patterns in adults, studies on sea urchins have generally not been linked to the evolution of chordates. However, recent molecular characterization of the oral ectoderm in their bilaterally symmetrical embryos is surprisingly helping to change this situation [24–26]. The oral–aboral polarity in a sea urchin embryo is initially discerned by zygotic *nodal* expression at the 32-cell stage, which results from maternal factors possibly controlled by a redox gradient [27]. Nodal activates the *lefty* gene and Lefty protein in turn controls *nodal* expression, resulting in shaping a *nodal–lefty* co-expression domain. In this domain, Nodal signalling activates *goosecoid*, *chordin*, *not*, *brachyury* and *foxa* genes directly or indirectly, and then differentiates the domain into the oral ectoderm [28,29]. All of these genes are involved in the dorsal formation of chordate embryos.

Recently, the role of Nodal signalling in the axial determination of amphioxus embryos was proposed [15]. If we take into account the similarity in early development up to the blastula stage between amphioxus and sea urchin embryos, then the gene regulatory network triggered by Nodal signalling for early regional specification may link amphioxus to outgroup sea urchins. Amphioxus embryos express the *lefty* gene as one of the earliest zygotic expressions on the one side at the 64- to 128-cell stage [15]. Interestingly, the *lefty*-expressing region seems to express *nodal*, and then *goosecoid* and *chordin* genes [21]. When Nodal signalling was blocked during cleavage, the *chordin* gene was not expressed in amphioxus embryos [15], suggesting it is a downstream target gene of Nodal signalling as observed in sea urchin embryos. These expression patterns during the blastula to early gastrula stage are very similar to those in the oral ectoderm of sea urchin embryos [15,25]. Based on these observations, in this study, we addressed the questions of how the earliest zygotic expression of *lefty* is controlled and of how the earliest *nodal–lefty* expression pattern provides the basis for dorsal structures in amphioxus. We show that a widely expanding blastopore and the resulting archenteron that comes into intimate contact with the external layer of the gastrula are key modifications to induce the dorsal structures from an ancestral blastula that could be comparable to blastulae of extant ambulacrarians (echinoderms + hemichordates) [30–32].

## 2. Results

### 2.1. Sperm entry site is not important for dorsoventral polarity in amphioxus embryos

**Figure 1.**
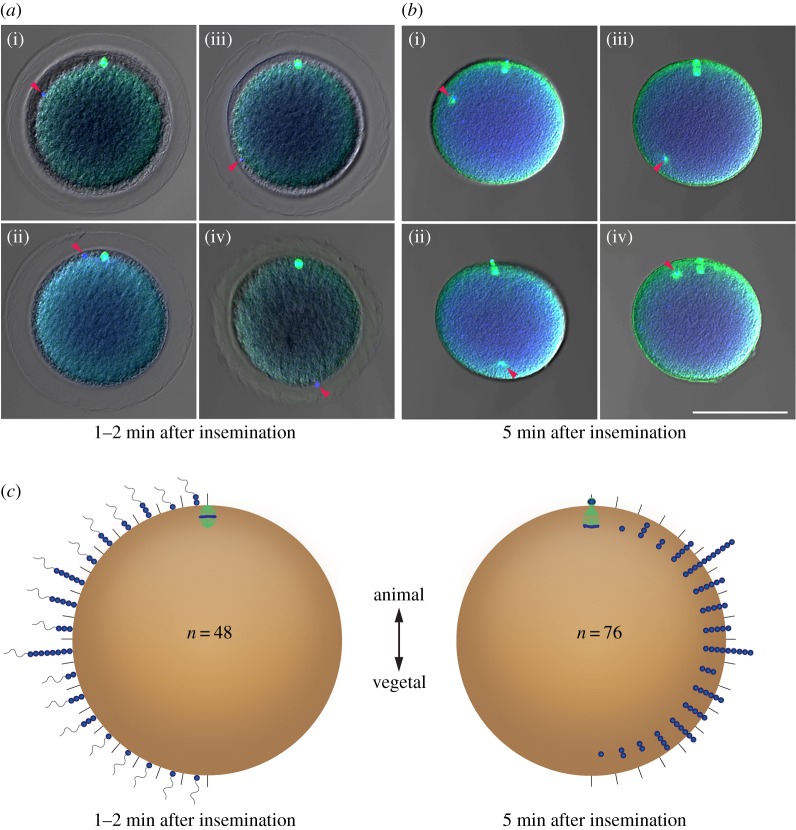
Sperm entry site and location of male pronucleus. (*a*(i–iv)) Four examples of sperm entry sites (arrowheads) at 1–2 min after insemination. (*b*(i–iv)) Varied locations of male pronucleus (arrowheads) at 5 min after insemination. (*c*) Histograms for sperm entry site (*n* = 48) and location of male pronucleus (*n* = 76) collectively showing each in a half circle; both show the number observed on/in sector divided by 10° in rendering image of 10 sections with 0.5 µm interval. Scale bar, 100 µm.

All studied anamniote vertebrate and ascidian eggs have sperm enter on the animal hemisphere, and in many cases the sperm entry point is important for determining embryonic axes [33], with some exceptions such as in zebrafish, in which the anteroposterior axis does not rely on fertilization [34,35]. The amphioxus egg is also believed to accept a sperm on the animal hemisphere, but differs from other chordates in the lack of conspicuous cytoplasmic rearrangement following sperm entry [16]. However, as the original observation of sperm entry sites was based on transmission electron microscopic analyses of only three eggs [16], we re-examined the entry point and probable trajectory of the male pronucleus by fixing eggs soon and 5 min after insemination (figure 1). Unlike the previous suggestion [16], sperm could enter anywhere on an egg with some preference for the region along the equator (*n* = 48), which is similar to the fertilization of sea urchin eggs [36]. At 5 min after insemination, the location of the male pronucleus also did not show any regional bias (*n* = 76). As male and female pronuclei met about 20 min after sperm entry on the one side of the animal hemisphere near the equator (electronic supplementary material, figure S1), the observed sperm entry points and the location of male pronuclei are inconsistent with the believed stereotypical movement of the male pronucleus. These observations suggest that the reorganization from radial to bilateral symmetry induced by the stereotypical behaviour of the male pronucleus and/or sperm-donated centrosome and microtubules extending from the centrosome as in *Xenopus* [37] and ascidians [9] is precluded as the mechanism of the axis determination in amphioxus.

### 2.2. Arp2/3 localization and asymmetrical *lefty–nodal* co-expression domain

**Figure 2.**
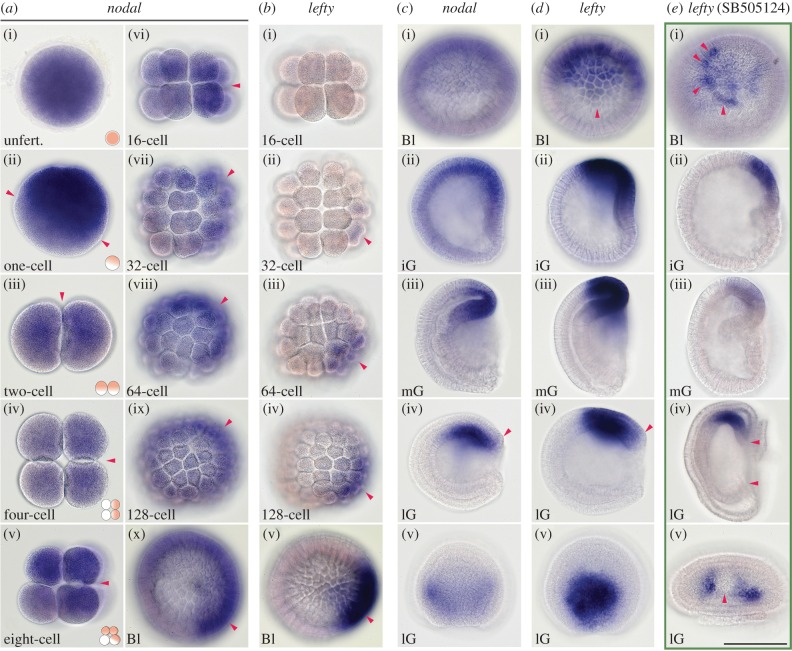
Maternal and zygotic expression of *nodal* and *lefty* genes. Animal pole to the top for all but *a*(iii–iv), *c*(i), *d*(i), *e*(i), *c*(v), *d*(v) and *e*(v). (*a*(i–ix)) Maternal expression of *nodal* gene showing gradual asymmetrical pattern (arrowheads). (*a*(x)) Initial zygotic expression of *nodal* in *lefty* expression domain (arrowhead). (*b*(i–v)) Onset of *lefty* zygotic expression at 32-cell stage on the one side (arrowhead). (*c*(i)*, d*(i)) Expression domain of *nodal* (*c*) and *lefty* (*d*) at blastula stage viewed from vegetal side. (*c*(ii–iv), *d*(ii–iv)) Lateral view (anterior to the left) of *nodal* (*c*) and *lefty* (*d*) expression from initial gastrula to late gastrula stage. Note attenuation of both gene expressions at very margin of blastopore (arrowheads). (*c*(v), *d*(v)) Dorsal view of *nodal* (*c*) and *lefty* (*d*) expression at late gastrula stage. (*e*(i–v)) Expression of *lefty* in SB505124-treated embryos. Note changes in expression pattern with strong expression spots in blastula (arrowheads in *e*(i)), shrunken blastopore and archenteron (arrowheads in *e*(iv)), and mid-dorsal deformation (arrowhead in *e*(v)). (*e*(i)) vegetal view, (*e*(ii­–iv)) lateral view and (*e*(v)) dorsal view. Bl, blastula; iG, initial gastrula; lG, late gastrula; mG, mid-gastrula. Scale bar, 100 µm.

**Figure 3.**
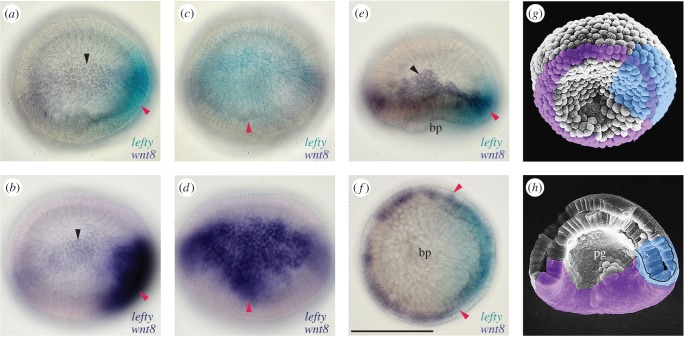
Expression of *lefty* and *wnt8* genes in single embryos. (*a*,*b*) Lateral view (animal pole to the top) of double WISH with *lefty* (red arrowhead) and *wnt8* (black arrowhead) probes at late blastula stage. Double colour precipitates (*a*) and single colour precipitates (*b*). (*c*,*d*) Future dorsovegetal view of the same samples as (*a*) and (*b*), respectively. Note apex pointing to vegetal pole (arrowheads). (*e*) Lateral view (animal pole to the top) of double WISH with *lefty* (red arrowhead) and *wnt8* (black arrowhead) at initial gastrula stage. (*f*) Blastopore view (dorsal to the right) of the same sample as (*e*). Note a wide mid-dorsal domain of *lefty* (arrowheads). (*g*,*h*) Scanning electron microscopic (SEM) montages showing expression domains of *lefty* (light blue) and *wnt8* (purple) at initial and mid-gastrula stage. Original SEM micrograph films from the late Dr R. Hirakow. bp, blastopore; pg, archenteron. Scale bar, 100 µm.

As *lefty* is one of the earliest genes expressed zygotically in *Branchiostoma floridae* as in sea urchin embryos [15], we re-examined the expression pattern of *lefty* from the one-cell stage to gastrula stage in *Branchiostoma japonicum*. No maternal expression of *lefty* was detected, and the initial zygotic expression was observed in several consecutive blastomeres at the 32-cell stage (figure 2). Unlike *B. floridae*, which initially retains *lefty* mRNA in the nucleus [15], however, *lefty* mRNA in *B. japonicum* was distributed in the cytoplasm from the beginning of its expression. The expression domain was expanded to occupy one of the four sectors on a hemisphere of a spherical embryo by the 128-cell stage (figure 2*b*(iv)). To identify the location of the *lefty* positive sector, double whole-mount *in situ* hybridization (WISH) with *lefty* and *wnt8* probes, the latter of which is expressed in a few cells' width along the equator at the blastula stage [38], was performed, and confirmed that the *lefty* positive domain expanded across the equator asymmetrically in terms of the animal–vegetal axis and the apical angle of the domain pointed towards the vegetal pole (figures 2*d*(i) and 3).

As Nodal signalling is responsible for initial *lefty* expression in sea urchins and vertebrates [30,39,40], we also re-analysed the expression pattern of *nodal* gene from the unfertilized egg to late gastrula stage in *B. japonicum*. Maternal mRNA of *nodal* was distributed evenly in unfertilized eggs (figure 2). After sperm fusion, *nodal* mRNA concentrated into the animal hemisphere and then displayed an asymmetrical pattern. During the first cleavage, the asymmetrically distributed mRNA was inherited evenly by halves of both daughter blastomeres, which were destined to become two blastomeres at the four-cell stage. The line of two highly concentrated blastomeres at the four-cell stage then divided into a line of two highly concentrated animal blastomeres and underneath two vegetal blastomeres that contain *nodal* mRNA in the same fashion as two-cell embryos. The other line of two animal blastomeres also contained the mRNA (figure 2*a*(ii–v)). This asymmetrical distribution of maternal *nodal* mRNA was traced until the 64- to 128-cell stage, and the *lefty* gene was activated at the 32-cell stage (figure 2*b*(ii)). During gastrulation, the expression domain of *nodal* was restricted within the *lefty*-expressing domain that was located across the margin of the blastopore (figure 2*c*(i,ii)). At the mid- and late gastrula stages, *nodal* expression in the outer layer became weakened and both *lefty* and *nodal* expressions were attenuated at the blastopore margin (figure 2*c*(iv),*d*(iv)). Our observations that the weak asymmetrical distribution of maternal *nodal* mRNA promotes the initial zygotic expression of *lefty* gene and, subsequently, zygotic *nodal* expression was restricted within the *lefty*-expressing domain may represent a *nodal–lefty* interaction known as a reaction–diffusion system in other deuterostomes [30,41,42].

To examine whether the Nodal signalling activates *lefty* expression in amphioxus embryos such as other deuterostomes, we treated embryos from the one- to 64-cell stage with a Nodal signalling inhibitor, SB505124 [43]. When treated, the expression of *lefty* did not disappear at the blastula stage, but the pattern of expression was affected (figure 2*e*(i–v)). The expression domain at the blastula stage showed various outlines, within which some strongly expressing cells were scattered (figure 2*e*(i)). This result suggests that Nodal signalling may be recovered soon after the removal of the drug and then the *lefty* gene is activated ectopically. In gastrulae, the *lefty* expression on the dorsal side was retained, but did not restore normal expression. Notably, some gastrulae displayed a groove or protrusion at the mid-dorsal region (figure 2*e*(v)). These observations support the idea that Nodal signalling based on the weak asymmetrical distribution of maternal *nodal* mRNA directly affects the asymmetrical expression of zygotic *lefty* gene in amphioxus embryos.

**Figure 4.**
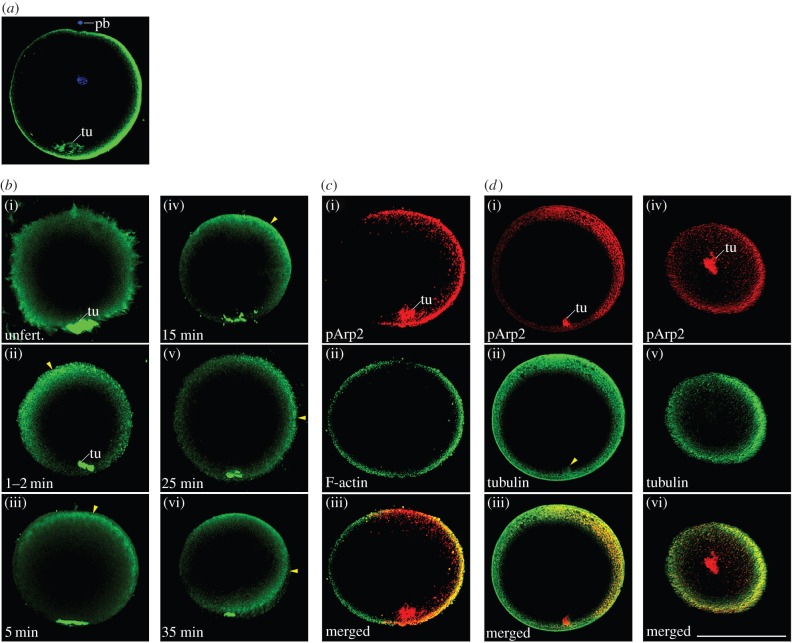
Distribution pattern of active Arp2/3 complex in unfertilized and fertilized egg and its co-localization with microtubules and actin filaments. Partial rendering lateral images near maximum diameter in all but (*d*(iv­–vi)), which are viewed vegetally near vegetal pole. (*a*) Eggs are oriented by the aid of polar body (pb) and/or vegetal tuft-like structure (tu). (*b*(i–vi)) Change in location of cortical pArp2 immunopositive signals after sperm fusion from ubiquitous in cortical region in unfertilized egg (*b*(i)) to on the one side of egg (*b*(vi)) passing through animal pole (*b*(iii,iv)) (arrowheads). (*c*(i–iii)) Co-localization of F-actin and pArp2 immunopositive signals in late fertilized egg. (*d*(i­–vi)) Co-localization of microtubules and pArp2 immunopositive signals in late fertilized egg. Note a tuft-like structure at the vegetal pole in immunostain for pArp2 and for tubulin (*d*(ii)). Scale bar, 100 µm.

**Figure 5.**
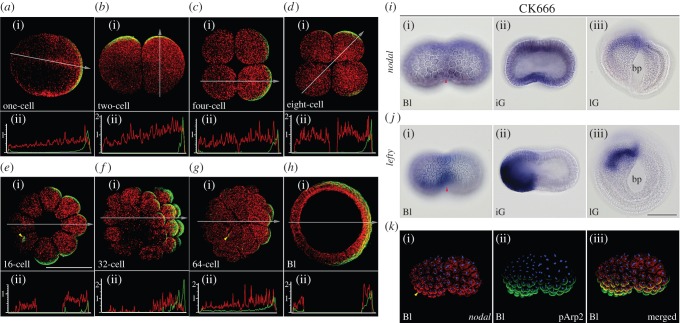
Distribution patterns of maternal *nodal* mRNA and Arp2/3 complex. (*a*(i)*–h*(i)) Maternal *nodal* mRNA (red) and pArp2 immunopositive signals (green) are co-localized from one-cell to blastula stage shown as partial rendering images near maximum diameter. Animal pole to the top in (*a*(i)), (*d*(i)) and (*h*(i)). (*a*(ii)*–h*(ii)) Relative fluorescent intensity curve at section denoted by white arrow that indicates direction of *x*-axis. Red for *nodal* and green for pArp2. Note a tuft-like immunopositive for anti-pArp2 antibody in a cell at 16- and 64-cell stage (arrowheads) (*e*(i),*g*(i)). (*i*(i–iii)) Expression pattern of *nodal* in CK666-treated blastula to late gastrula stage. (*j*(i–iii)) Expression pattern of *lefty* in CK666-treated blastula to late gastrula stage. Note expression at median furrow (arrowheads) and a half of embryo in both genes. (*k*(i–iii)) Co-localization of pArp2 immunopositive signals and *nodal* mRNA in CK666-treated blastula. Bl, blastula; bp, blastopore; iG, initial gastrula; lG, late gastrula. Scale bar, 100 µm.

Further, to understand the mechanism causing the initial asymmetrical distribution of maternal *nodal* mRNA, we next examined whether the remodelling of cortical cytoskeletons in fertilized eggs could be responsible for this asymmetrical distribution of maternal *nodal* mRNA, as amphioxus eggs do not show any conspicuous ooplasmic rearrangement and/or microtubule formation from the centrosome unlike the situation in ascidian and *Xenopus* eggs [9,37]. The active form of Arp (actin-related protein) 2/3 complex is a good candidate for analysing the remodelling of the organization of cortical actin filaments as it functions in nucleation and branching of actin filaments to activate cytoskeletal reorganization [44–46]. We found that immunostainings for phosphorylated Arp2 (pArp2), which composes the active form of the Arp2/3 complex, in unfertilized and fertilized eggs displayed a movement of the immunopositive signals to one side of the egg via the animal pole in the cortical region (figure 4*a,b*). The asymmetrical distribution of the active Arp2/3 was co-localized with tubulin and F-actin immunopositive signals in fertilized eggs, as well as with tubulin in cleavage embryos (figure 4*c*,*d* and electronic supplementary material, figure S2). We also found a tuft-like immunopositive structure at the vegetal pole in unfertilized and fertilized eggs, which was traceable at least until the 64-cell stage (figures 4 and 5*e*(i),*g*(i)).

We then performed fluorescent WISH with *nodal* probe and immunoreaction with an anti-pArp2 antibody for fertilized eggs to blastulae (figure 5). The fluorescent WISH signals reproduced the asymmetrical expression pattern depicted by the ordinal WISH (figure 2) and the asymmetrical distribution of maternal *nodal* mRNA was co-localized with the active Arp2/3 complex. To examine whether active Arp2/3 localization actually affects the distribution of maternal *nodal* mRNA, we disturbed Arp2/3 function with CK666, a drug that blocks the conformational change of Arp2/3 [47], soon after fertilization until the two-cell stage. Most of the treated embryos developed into blastulae in the shape of a groundnut hull (figure 5*i*(i),*j*(i)). In these blastulae, immunopositive signals of anti-pArp2 antibody were detected in the region where *nodal* and *lefty* genes were expressed ectopically (figure 5*k*). These results suggest that the activation of Arp2/3 complex affects the distribution of maternal *nodal* mRNA.

### 2.3. Dorsal-specific genes are downstream genes expressed within *lefty–nodal* co-expressing domain

**Figure 6.**
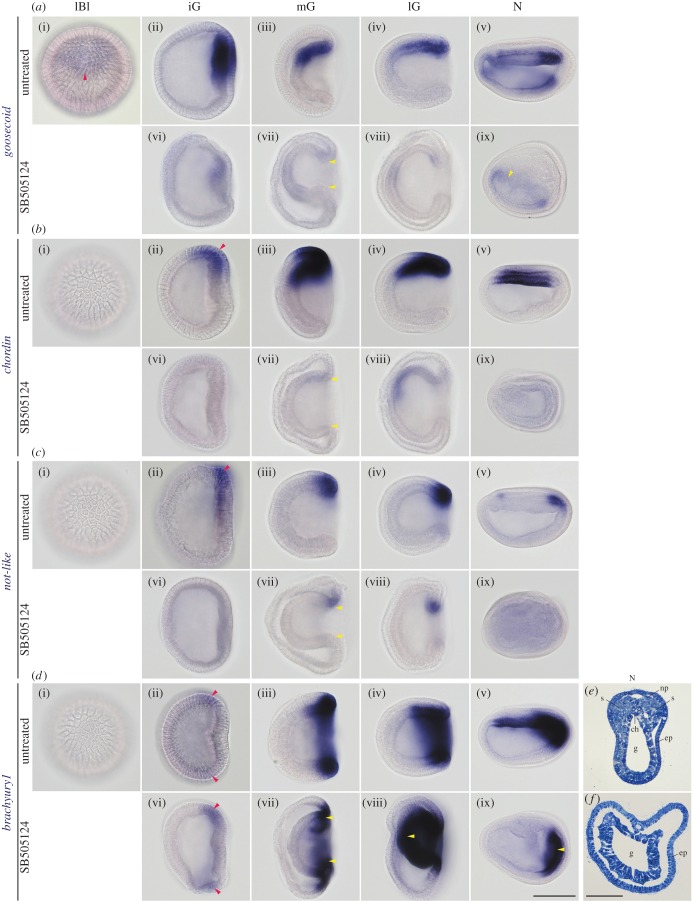
Disappearance of downstream target gene expressions in embryos treated with Nodal signalling inhibitor (SB505124). All lateral view (anterior to the left) except late blastula stage. Initial expressions are denoted by red arrowheads. (*a*(i–ix)) Zygotic expression of *goosecoid* in untreated (*a*(i–v)) and short-term-treated (*a*(vi–ix)) embryos. (*b*(i–ix)) Zygotic expression of *chordin* in untreated (*b*(i–v)) and short-term-treated (*b*(vi–ix)) embryos. (*c*(i–ix)) Zygotic expression of *not-like* in untreated (*c*(i–v)) and short-term-treated (*c*(vi–ix)) embryos. (*d*(i–ix)) Zygotic expression of *brachyury1* in untreated (*d*(i–v)) and short-term-treated (*d*(vi–ix)) embryos. (*e*) Transverse section of untreated neurula showing differentiating dorsal structures. (*f*) Transverse section of short-term-treated neurula showing lack of dorsal structures. Note shrunken blastopore and archenteron (arrowheads), expanded expression of *brancyury1* in archenteron (arrowhead in *d*(viii)) and retained non-mid-dorsal expression in treated embryos (arrowheads in *a*(ix) and *d*(ix)). ch, notochord; ep, epidermis; g, gut; iG, initial gastrula; lBl, late blastula; lG, late gastrula; mG, mid-gastrula; N, neurula; np, neural plate; s, somite. Scale bar, 100 µm for all, but 50 µm for (*e*) and (*f*).

Gastrulation in amphioxus embryos starts as flattening of the vegetal hemisphere as in sea urchin embryos, but is wider than the latter, expanding to the equatorial region (figure 6*a*(ii)). The invagination process occurred at the equator where *wnt8* was expressed. Through this gastrulation, the *lefty*–*nodal* co-expression domain was bent at the blastopore lip and finally the internalized domain became underlying the external domain (figures 2*c*(ii,iii), *d*(ii,iii) and 3*g*,*h*). During the bending at the blastopore lip, *goosecoid*, *chordin*, *not-like* and *bachyury1* genes were expressed within the *lefty*–*nodal* co-expressing domain (figure 6 and [21]). Among these four genes, *goosecoid* was the earliest gene to be expressed. Its initial expression domain at the late blastula stage corresponded with the *lefty* domain (figure 6*a*(i)). The expression of *chordin* and *not-like* started at the initial gastrula stage again corresponding with the *lefty–nodal* co-expression domain (figure 6*b*(ii),*c*(ii)). A T-box gene, *brachyury1*, differed from the above-mentioned genes in terms of expression. This gene was initially expressed along the equatorial region within the domain of *wnt8*. When the expression of *wnt8* attenuated at the mid-dorsal region (figure 3*f*), the mid-dorsal expression of *brachyury1* was retained and expanded anteriorly in accordance with the axial expansion (figure 6*d*(ii–­v)).

To examine whether these genes are direct or indirect downstream target genes of the Nodal signalling, embryos were treated with SB505124 from the one- to 64-cell stage or until the stages at fixation, and the expression of *goosecoid*, *chordin*, *not-like* and *brachyury1* was observed. All of these genes disappeared or were reduced in their expression in treated embryos (figure 6 and electronic supplementary material, figure S3), suggesting that these genes are regulated under Nodal signalling similar to as in the oral ectoderm of sea urchin embryos [28,48]. Interestingly, the treated embryos reduced the diameter of the blastopore, and the archenteron did not touch the outer layer (figures 2*e*(iv) and 6*a*(vii), *b*(vii), *c*(vii), *d*(vii)), as seen in ambulacrarian embryos [28,32]. In treated neurulae, the axial expression of *goosecoid*, *chordin*, *not-like* and *brachyury1* was not detected. Histological sections of treated neurulae lacked the notochord and neural plate beneath the epidermis, consistent with the absence of the dorsal gene expression (figure 6*e*,*f*). Interestingly, however, *goosecoid* expression in the anterior gut and *brachyury1* expression at the blastopore margin and then at the tailbud seemed to be normal (figure 6*a*(ix),*d*(ix)), suggesting different control mechanisms from those of the dorsal axis expression domains.

### 2.4. Early zygotic expression of *bmp2/4* occurs only in invaginating archenteron with a dorsoventral gradient

**Figure 7.**
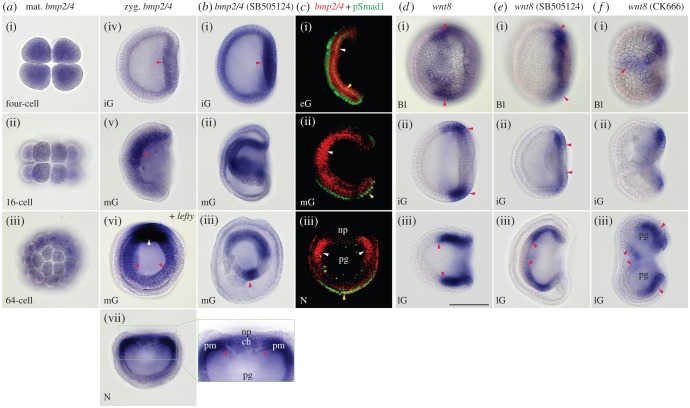
Expression pattern of *bmp2/4* and *wnt8* in embryos untreated and treated with inhibitors. (*a*(i–vii)) Maternal and zygotic (red arrowheads) expression of *bmp2/4* in untreated embryos. (*a*(vi)) Double WISH for *bmp2/4* (red arrowheads) and *lefty* (white arrowhead) showing the direction of *bmp2/4* gradient. Blastopore view. (*a*(vii)) Blastopore view of early neurula showing clear contrast of expression between paraxial mesoderm and axial structures (red arrowheads) in its magnification. (*b*(i–iii)) Expression pattern of SB505124-short-term-treated embryos showing enhanced and ectopic expression (arrowhead in *b*(iii)). (*c*(i)) Co-localization of *bmp2/4* mRNA (white arrowhead) and anti-pSmad1 immunopositive signals (yellow arrowhead) at early gastrula (partial rendering image in left lateral view). (*c*(ii–iii)) Opposed distributions of *bmp2/4* mRNA and anti-pSmad1 immunopositive signals at mid-gastrula (*c*(ii): partial rendering image in left lateral view) and early neurula (*c*(iii): partial rendering image viewed from blastopore). Note nuclear accumulation of pSmad1 in ventral cells (yellow arrowheads). Green signals in archenteron are non-specific. (*d*(i–iii)) Expression of *wnt8* in untreated embryos (lateral view). (*e*(i–iii)) Expression of *wnt8* shifted towards vegetal pole (arrowheads in *e*(i–ii)) and its expansion in archenteron (arrowheads in *e*(iii)) in SB505124-short-term-treated embryos (lateral view). (*f*(i–iii)) Expression pattern of *wnt8* in CK666-treated embryos showing bifurcated expression (arrowheads in *f*(i)) and double gastrulation (arrowheads in *f*(iii)). Bl, late blastula; ch, notochord; iG, initial gastrula; lG, late gastrula; mG, mid-gastrula; N, neurula; np, neural plate; pg, archenteron; pm, paraxial mesoderm. Scale bar, 100 µm.

Bmp signalling functions in dorsoventral patterning with its antagonist Chordin in bilaterians [49,50]. However, the expression pattern of the bmp (*dpp*) gene is variable among bilaterians, especially among deuterostomes. In sea urchin embryos, the *bmp2/4* gene is promoted by Nodal signalling on the oral side [24]. Nodal signalling also activates the *chordin* gene on the same side [48], which is peculiar compared with other bilaterians that express these two genes on opposite sides. Early expression patterns of *bmp2/4* in amphioxus species have been reported, but they are not consistent between species or suggest interspecific differences [38,51]. We thus re-examined the expression pattern of *bmp2/4* in *B. japonicum*. Maternal signals were detected ubiquitously and zygotic signals were initially discernible at the centre of the vegetal plate at the initial gastrula stage (figure 7*a*). During gastrulation, expression was extended within the invaginating archenteron producing a dorsoventral gradient with the highest expression on the future dorsal side (figure 7*a*(v,vi)). The attenuation of expression at the mid-dorsal region was delayed from the onset of *chordin* expression, probably reflecting the timing of the accumulation of Chordin protein. Once the expression of *bmp2/4* disappeared from the mid-dorsal region, the boundary between the strongest expression at the paraxial mesoderm and the *bmp2/4*-free axial mesoderm became sharp (figure 7*a*(vii) and [51]).

As expression of *bmp2/4* was gradually stronger towards the *nodal*-expressing side, we again examined the relationship between Nodal signalling and *bmp2/4* expression by blocking the signal with SB505124. Ubiquitous expression (probably maternal) was retained until initial gastrulation, and zygotic expression seemed to be enhanced with an ectopic expression in the ventral region of the archenteron (figure 7*b*).

As the Bmp2/4 protein expressed on the oral side in sea urchin embryos is transferred with Chordin as a shuttle protein to the aboral side where Bmp2/4 signalling can function [48], we also examined the distribution of phosphorylated Smad1/5/8 (pSmad1/5/8) [52] with an antibody to pSmad1, which is a transcription factor specific to Bmp2/4 signalling. Immunopositive signals were observed in the invaginating archenteron at the early gastrula stage, which were co-localized with the expression of *bmp2/4* (figure 7*c*). Interestingly, however, pSmad1/5/8 immunoreactions were detected in ventral ectoderm at the late gastrula and neurula stages, where expression of *bmp2/4* was weak or absent (figure 7*c*(ii,iii)). The spatial relationship between *bmp2/4* expression and pSmad1/5/8 in late gastrulae and neurulae is again comparable to their counter distributions observed in sea urchin embryos [48].

### 2.5. Expression domain of *wnt8* is affected by Nodal signalling inhibitor

The initial equatorial expression of *wnt8* is important, because this corresponds with the blastopore margin in amphioxus embryos. As we obtained gastrulae with a small blastopore when treated with the Nodal inhibitor (figures 2 and 6), we examined whether *wnt8* expression is affected by the treatment of SB505124. When embryos were treated from the one- to 64-cell stage, the circular expression of *wnt8* was apparently shifted towards the vegetal pole (figure 7*d*,*e*). Correspondingly, the diameter of the blastopore was reduced, and invaginating archenteron was separated from the outer layer as in ambulacrarian gastrulation. In the archenteron, the expression of *wnt8* was expanded anteriorly (figure 7*d*(iii),*e*(iii)), which also induced a correspondingly expanded expression of *brachyury1* in the archenteron (figure 6*d*(viii)). The groundnut hull-shaped blastula treated with CK666 produced double gastrulation. These embryos showed a bifurcated expression pattern of *wnt8* (figure 7*f*). These results suggest that the initial *wnt8* expression domain is tightly related to the gastrulation and that early Nodal signalling links to the patterning of the *wnt8* expression domain.

## 3. Discussion

We analysed the mechanism underlying amphioxus dorsal formation primarily by using drugs that disturb the Nodal signalling and actin-related protein 2/3 complex. We showed that amphioxus initiates dorsal formation in a manner similar to the oral ectoderm specification in sea urchin embryos, although the former is initiated by maternal Nodal signalling, whereas the latter is by zygotic signalling. The key feature for establishing dorsal structures from a sea urchin-like asymmetrical expression of Nodal signalling could be the expansion of the blastopore towards the equator in amphioxus embryos.

### 3.1. Establishment of asymmetrical co-expression domain of *nodal* and *lefty*

Unlike other chordate species, as the amphioxus egg does not show conspicuous ooplasmic rearrangement after sperm fusion [16], the localization of mitochondria that accompany the male pronucleus was proposed as the causative event for breaking the egg's radial symmetry [15]. However, our observations of sperm entry points were not consistent with the stereotypical trajectory of the male pronucleus as has been suggested previously [16], but instead similar to the pattern observed in sea urchins [36]. A redox gradient with asymmetrical distribution of mitochondria has also been proposed as an initial regulator for the determination of the oral side in sea urchin embryos [53–55]. However, even in sea urchins, there is still no consensus about the initial regulator that breaks the egg's radial symmetry [26]. Our observation of mitochondrial distribution visualized with MitoTracker (Molecular Probes, OR) in fertilized amphioxus eggs also did not show any significant asymmetrical pattern (electronic supplementary material, figure S4).

We confirmed that Nodal signalling is a key player, as suggested previously [15], for establishing the zygotic expression domain of the *nodal* gene that is regulated by Lefty–Nodal interaction as seen in sea urchin embryos, but differing from the latter by having maternally supplied *nodal* mRNA in amphioxus embryos. As the initial step for the radial symmetry break, the asymmetrical distribution of maternal *nodal* mRNA was produced by a remodelling of cortical cytoskeletons, whose process was visualized for the first time by the immunolabelling of the active Arp2/3 complex and WISH for *nodal* transcripts (figure 5). The active Arp2/3 complex is known to promote the remodelling of cortical cytoskeletons receiving various signals in a wide range of animal cells including eggs [56,57]. The dynamics of cortical cytoskeletons mediated by the Arp2/3 complex is also tightly linked to the molecular dynamics in cell membrane [58]. In an ascidian, the segregation of *not* mRNA that initiates mesodermal fate from the mesendoderm cell is cued by the cell membrane localization of phosphatidylinositol (3,4,5)-bisphosphate (PI(3,4,5)P_3_), which is regulated by maternal phosphatidylinositol 3-kinase (PI3K) [11]. Although we still lack evidence for direct interactions between cortical cytoskeletons and *nodal* mRNA, disturbance of the proper distribution of the Arp2/3 and of shaping the *lefty–nodal* expression domain by the perturbation with CK666 suggests their intimate relationship. Germplasm-related mRNAs, *vasa* and *nanos*, were reported to be localized at the vegetal pole in amphioxus eggs [59] and *tbrain* (= *eomesodermin*) mRNA showed the same expression pattern (electronic supplementary material, figure S5). Their distribution pattern implies that the tuft-like structure that is immunopositive to the anti-pArp2 antibody found in this study has some role in the localization of mRNAs, which may support interaction between *nodal* mRNAs and the active Arp2/3 complex. As the remodelling of cytoskeletons is regulated by complicated mechanisms [58,60], our study is the first step to elucidate the mechanisms underlying the initiation of dorsal formation in amphioxus embryos.

### 3.2. Co-expression of *bmp2/4* and *chordin*

Bmp (Dpp) and Chordin are the representative molecules of the dorsoventral polarity in bilaterians [49,50]. Curiously, sea urchin embryos express these two genes on the oral (ventral) side promoted by Nodal signalling [48]. However, Bmp2/4 is blocked in its function on the oral side, but is transferred to the aboral (dorsal) side, where Bmp2/4 signalling becomes active. A similar co-expression has also been reported in the cnidarian species *Nematostella vectensis* [61,62], and it has been suggested that the co-expression of Bmp and Chordin is possibly a plesiomorphic character found in both cnidarians and bilaterians [48]. Interestingly, the *bmp2/4* expression in amphioxus embryos showed a gradient with the strongest expression at the mid-dorsal region, where *lefty* and *nodal* genes were continuously expressed and activated the *chordin* gene. Thus, amphioxus is another example of the co-expression of *bmp2/4* and *chordin* genes. Further, active Bmp2/4 signalling detected by the immunoreaction for pSmad1/5/8 was localized in the ventral ectoderm. These opposed gradients between *bmp2/4* expression and active signalling are comparable to those of sea urchin embryos [48,63]. However, unlike sea urchin embryos, the expression of *bmp2/4* disappeared from the *chordin*-expressing domain and resulted in the chordate pattern, in which the notochord develops from the *bmp2/4*-free mid-dorsal archenteron and the neural plate develops from the overlying ectoderm.

### 3.3. Expression of *wnt8* and gastrulation pattern

Early amphioxus development is similar to that of ambulacrarians until the blastula stage, displaying a single-layered spherical coeloblastula. Amphioxus embryos initiate gastrulation as the vegetal plate again similar to as in ambulacrarians [64,65], but the blastopore margin comes to the equator, where *wnt8* is expressed as the initial sign for gastrulation [38], resulting in a large blastopore. Sea urchin embryos express multiple wnt genes in the future vegetal plate [66], where the *brachyury* gene is expressed circularly surrounding the vegetal pole and prefigures the blastopore margin [67]. As nuclear accumulation of β-catenin occurs vegetally, sea urchin *brachyury* was regarded to be regulated by Wnt/β-catenin signalling [67]. In amphioxus embryos, the expression of *brachyury1* gene occurs within the *wnt8* domain soon after the equatorial expression of *wnt8* gene [38,68]. The nuclear accumulation of β-catenin in amphioxus occurs ubiquitously in blastulae [19,20] and the circular expression of *wnt8* may reinforce the level of nuclear β-catenin and trigger the earliest expression of *brachyury1* in amphioxus, as has been suggested for sea urchin embryos [67,69].

Interestingly, SB505124-treated amphioxus resulted in gastrulae with a small blastopore, in which the circular expression of *wnt8* and *brachyury1* was shifted towards the vegetal pole (figures 6*d*(vi) and 7*e*). This result also suggests an unexpected linkage between Nodal signalling and *wnt8* gene in amphioxus embryos. In sea urchin embryos, Nodal and Wnt signallings are mutually antagonistic and the *nodal* expression domain on the oral side does not overlap with that of multiple wnt genes on the vegetal side [70]. In contrast, the circular expression of *wnt8* in amphioxus crossed the *lefty–nodal* expression domain (figure 3). During the blastula to early gastrula stage in amphioxus embryos, Wnt-signalling antagonists, two *dkks* encoding Dickkopf proteins and two genes encoding secreted Frizzled-like proteins, are expressed animally and vegetally [21], thereby sandwiching the *wnt8*-expressing ring. These proteins may restrict *wnt8* expression to the equator in amphioxus embryos. The difference in possible interactions of Wnt signalling with Nodal signalling, as well as with Wnt antagonists found between amphioxus and sea urchin embryos might arise from the fact that a single *wnt8* gene is involved in the former and multiple wnt genes in the latter [66] at the initial stage.

As a result of the equatorial expression of *wnt8* gene in amphioxus blastulae, the asymmetrical *lefty–nodal* expression domain is folded at the blastopore margin (figures 2*c*(ii,iii), *d*(ii,iii) and 3). The internalized archenteron soon attaches itself to the outer layer (ectoderm) and thus the *lefty–nodal* expression domain is located on the one side of the blastopore. In the *lefty–nodal* expression domain, genes that promote dorsal characterization, which are represented by *goosecoid*, *chordin*, *not-like* and *brachyury1*, are activated with *goosecoid* being expressed first at the late blastula stage. In sea urchin embryos, all of these genes function to differentiate the oral ectoderm [25]. Orthologous genes of wnt and nodal in vertebrates such as zebrafish and *Xenopus* embryos also play important roles in initial dorsoventral patterning and mesoderm formation, and similar expression patterns of these genes at the blastula stage are found in these vertebrates and in amphioxus. In particular, expression patterns of these genes in amphioxus and *Xenopus* blastulae are extremely similar [71,72]. However, mechanisms to establish similar expression patterns are diversified. For example, in zebrafish 3′ UTR of *squint* mRNA, Y box-binding protein 1 and microtubules are important for the localization of *squint* transcripts [73,74]. In contrast, in *Xenopus*, the gradation of nodal-related gene expression is controlled by maternal Wnt/β-catenin and Vg1. An array of microtubules is also important for localizing Wnt/β-catenin signalling, but not for direct localization of nodal-related mRNA unlike zebrafish [3]. Thus, it is difficult to speculate on what common denominator gave rise to the chordate body pattern, although molecules such as Wnt-signalling antagonists and Tgf-β family members are commonly thought to be involved. The formation of an eccentric gradient governed by Nodal signalling in conjunction with gastrulation, which could be provided by various manners actually seen in extant chordates and has been examined experimentally in zebrafish [75], may be a common feature among these chordates. As Nodal signalling is now known to be very important for the initial body patterning in various animals [76,77], the role of the Nodal signalling in the dorsoventral patterning in amphioxus cannot directly support a homology to that in *Xenopus* or more broadly anamniote embryos. It may be a kind of deep homology. To better understand this issue, we need to know the details of the mechanisms of interaction between nodal mRNA/protein and interactive molecules in amphioxus.

Genes important for notochord development such as *brachyury* and *foxa* in chordates are also expressed at the blastopore margin or in the archenteron in many animals including amphioxus [38,68,71,78–80]. Expression of these two genes at the blastopore margin in cnidarians [81,82] and protostomes [83–85] seems to be related to oral development, and even in protostomes that develop the mouth independently of the blastopore, *brachyury* and *foxa* are expressed at the future oral region and promote oral differentiation independently of that of the blastopore margin [78,86]. Stomodeal development in sea urchin embryos shares this feature [87]. The upstream gene regulation for *brachyury* and *foxa* in the oral region is different from that at the blastopore margin and archenteron, and the regulation of *brachyury* expression by Wnt signalling at the blastopore margin may be ancestral, because cnidarians retain this regulation [88], although this is not common in protostomes [89]. Even in amphioxus embryos, the mid-dorsal expression of *brachyury1* is regulated independently of that at the blastopore margin; the former is under Nodal signalling and the latter probably under Wnt signalling as seen in sea urchin embryos [70]. This distinction in regulatory mechanisms is probably retained in vertebrates as a variety of derived machinery for dorsoventral mesodermal patterning [71,80].

**Figure 8.**
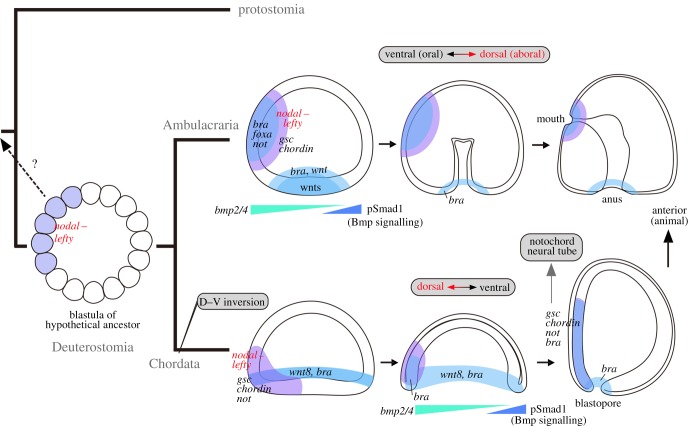
Phylogenetic relationship between chordate dorsal structures and ambulacrarian oral ectoderm. Shared molecular patterning of ambulacrarian oral ectoderm and amphioxus dorsal specification consistent with dorsoventral inversion hypothesis occurred in chordate lineage. The last common ancestor of deuterostomes is parsimoniously supposed to have passed through a coeloblastula with asymmetrical *nodal–lefty* expression, but it remains unknown whether this blastula type is ancestral or a derived character. Ambulacrarian pattern is adapted from [25,66,67].

In this study, we showed multiple lines of similarity in the early embryonic development and gene regulation between amphioxus and sea urchins. As chordates use a gene regulatory network that promotes the oral differentiation in sea urchin embryos for developing the notochord, they needed to acquire a new mechanism to open mouths. Consistently, amphioxus larvae open their unique mouth under a mechanism distinct from that of ambulacrarians and the other chordates also have a mouth different from that of ambulacrarians and amphioxus [90]. Our present observations are also compatible with the dorsoventral inversion proposed to have occurred in the last common ancestor of chordates [50]. This study suggests that extant amphioxus retains evidence of this dorsoventral inversion in its development. The last common ancestor of deuterostomes may have developed passing through a coeloblastula stage, and in the chordate lineage the blastopore margin expanded towards the equator, which modified the ancestral oral region to be located on the one side of the blastopore margin, from which chordate dorsal structures were newly formed (figure 8). We propose that this blastopore expansion was critical in the chordate ancestor acquiring the dorsal structures.

## 4. Material and methods

### 4.1. Experimental animals

Amphioxus embryos were obtained from a laboratory colony of *Branchiostoma japonicum* maintained at the Marine Station of Kumamoto University during breeding seasons [91].

### 4.2. Detection of sperm entry point and male pronucleus

Fertilized eggs were fixed with chilled methanol at 1–2 and 5 min after insemination and stored at −20°C until use. Specimens were rehydrated through a graded methanol series and transferred into phosphate-buffered saline (PBS), in which DNA was stained with Hoechst (Life Technologies, CA) at 2 µg ml^−1^. After washing, specimens were passed through a graded glycerol/PBS up to 80%. Each specimen was oriented with the aid of the polar body and the signal of the male pronucleus, and a partial rendering image was obtained by confocal laser scanning microscope (LSM; ECLIPS C1, Nikon, Japan) scanning 10 sections with 0.5 µm intervals to cover the polar body and male nucleus at the maximum diameter of each egg.

### 4.3. Preparation of RNA probes

Embryos at the neurula stage with seven to nine somites (12 h postfertilization (hpf) at 24°C) were used for constructing template first strand cDNA by using ISOGEN (Nippon Gene, Japan) for total RNA isolation, and SMARTer RACE cDNA amplification kit (Clonetech, CA) and PrimeScript II kit (Takara, Japan). A list of the studied genes and primer sets for PCR amplification of fragments, including the coding region of *brachyury1*, *chordin*, *goosecoid, lefty* and *nodal* genes, are shown in the electronic supplementary material, table S1. PCR products of expected size were subcloned into pGEM-T Easy vector (Promega, WI) and sequenced from both ends to confirm identity. Digoxigenin- or fluorescein-labelled antisense riboprobes were synthesized with SP6, T3 or T7 RNA polymerase by using each linearized vector containing a cloned gene fragment as template (Roche Applied Science, Germany).

### 4.4. Whole-mount *in situ* hybridization

Embryos were fixed with freshly prepared 4% paraformaldehyde (PFA) in 0.5–1.0 M NaCl and 0.1 M 3-(*N*-morpholino) propanesulfonic acid buffer (pH 7.5) at 4°C overnight. Cleaving embryos were fixed with 8% PFA in the same buffer with 1.0–1.5 M NaCl. Fixed embryos were washed with 50% ethanol and then stored in 75% ethanol at −20°C until use. Dechorionation of unhatched embryos was carried out with fine tungsten needles. WISH was performed as described previously [79] with some modifications. The duration of prehybridization and hybridization was extended to 4 h at 50°C and to 16 h at 60°C, respectively. During washing, a treatment with RNase (50 µg ml^−1^) was carried out for 30 min. Blocking prior to immunoreaction was performed with a blocking solution (BS) containing 0.15 M NaCl, 1% (w/v) blocking reagent (Sigma-Aldrich, MO), and 10% heat-inactivated sheep serum (Cosmo Bio, Japan) in 0.1 M Tris–HCl (pH 7.4) for 1 h at room temperature. The BS was also used for immunoreaction solution. Visualization was performed incubating specimens in the solution containing substrate at 30°C. Double WISH was performed according to Yu *et al.* [21] with some modifications. After hybridization, purple colour was generated with NBT/BCIP (Roche Applied Science, Germany) at room temperature for 6 h. Alkaline phosphatase activity of the antibody for the first target was inactivated in 0.1 M glycine–HCl (pH 2.2) for 5 min and twice in 0.1 M glycine–HCl (pH 2.2) with 0.1% Tween 20 for 10 min. Samples were thoroughly washed in PBS with 0.1% Tween 20, blocked in the BS for 30 min, and then reacted with an antibody against the second target. Cyan colour was generated by BCIP at 30°C for 6 h.

### 4.5. Double fluorescent labelling by whole-mount *in situ* hybridization and immunohistochemistry

Detection of *nodal* mRNA and the active form of Arp2/3 complex, as well as of *bmp2/4* mRNA and phosphorylated Smad1 (pSmad1) was performed. Riboprobes were first detected by TSA Plus cyanine 3/fluorescein system (PerkinElmer, MA) as described previously [11]. After the completion of WISH, the specimens were subjected to immunostaining. The antibodies were anti-Arp2 (Thr237/Thr238) phospho-specific antibody (AP3871, ECM Biosciences, KY) for detecting active Arp 2/3 complex and anti-phospho-Smad1 (Ser463/465) antibody (06-702, Merck Millipore, Germany) for Bmp signalling. Immunoreactions were performed as described previously [92] at 1 : 100 dilutions for both antibodies. The secondary antibody was anti-rabbit IgG antibody labelled with Alexa Fluor 488 (Life Technologies, CA) at 1 : 400 dilution. DNA was stained with Hoechst at 2 µg ml^−1^ while washing the secondary antibody. Partial rendering images were obtained by using the LSM.

### 4.6. Fluorescent immunostaining

To observe actin filaments or microtubules with the active Arp2/3, double immunostainings were performed with the above antibody and anti-human F-actin (FUMCA358GT, Funakoshi, Japan) or anti-human α-tubulin (CLT9002, Cedarlane, Canada) antibody at 1 : 100 dilutions as described previously [92]. The secondary antibodies were anti-mouse IgG antibody labelled with Alexa Fluor 488 for the anti-F-actin and -tubulin antibodies and anti-rabbit IgG antibody with Alexa Fluor 555 for the anti-pArp2 antibody. Partial rendering images were obtained by using the LSM.

### 4.7. Pharmacological treatments

To block Nodal signalling, embryos were treated with SB505124 (S4696, Sigma-Aldrich, MI) soon after fertilization. In long-term treatment, embryos were allowed to develop in Millipore filtered seawater (MPFSW) at 50 µM SB505124 concentration until fixation at the mid-gastrula stage (6 hpf at 25°C), otherwise (short-term at 75 µM) triple washings were performed during the 32- to 64-cell stage (135–155 min postfertilization (mpf) at 25°C), and then embryos were also allowed to develop until fixation at the blastula (200 mpf), initial gastrula (4.5 hpf), mid-gastrula (6 hpf), late gastrula (7.5 hpf) and neurula (12–13 hpf) stages. For perturbing the active form of Arp2/3 complex, embryos were treated with CK666 (SML0006, Sigma-Aldrich) at 400 µM in MPFSW soon after fertilization and washed thoroughly three times at the two-cell stage (1 hpf at 25°C) in MPFSW. Embryos were allowed to develop until fixation at the blastula (200 mpf), initial gastrula (4.5 hpf), mid-gastrula (6 hpf) and late gastrula (7.5 hpf) stages. Treated embryos and those reared in MPFSW with the same volume of dimethyl sulfoxide (DMSO) added were fixed under the same protocol as for WISH specimens.

### 4.8. Semi-thin plastic sections

SB505124-treated and DMSO control embryos fixed for WISH at the neurula stage were embedded in Epon 812 resin as described previously [93]. The resin blocks were trimmed and serially sectioned at 1 µm thickness and stained with toluidine blue.

### 4.9. Image data processing

JPEG- or TIFF-format digital images were obtained by an optical microscope (TE2000, Nikon, Japan) and LSM. All images were visually optimized and edited with Adobe Photoshop and Illustrator CS6 (Adobe, CA).

## Supplementary Material

Table S1

## Supplementary Material

Figures S1, S2, S3, S4, and S5

## Data accessibility

DNA sequences: GenBank accessions *bmp 2/4* (AF206325), *brachyury1* (LC127054), *chordin* (LC127055), *goosecoid* (LC131329), *lefty* (LC127056), *nodal* (AB097411), *not-like* (LC127057) and *wnt8* (AF206500).
