# Supplementary material for: Acquisition of the dorsal structures in chordate amphioxus
Source: Open Biol. 2016 Jun 15;6(6):160062. doi: 10.1098/rsob.160062 (PMC4929940; doi:10.1098/rsob.160062)
Supplement: Table S1 [file rsob160062supp1.docx]

**Table S1.** List of genes and primer sets used in this study

| **Gene** | **Primer set** | **Accession number** | **Reference** |
| --- | --- | --- | --- |
| *bmp 2/4* | --- | AF206325 | Yasui et al. (2001)[34] |
| *brachyury1* | 5’-GTTCKGCGGARACSATGAAG-3’  5’-GGTGGAGTCATRGGGCTCCA-3’ | LC127054 | Present study |
| *chordin* | 5’-GAAAGACACCGTACCAGGAT-3’  5’-CTTCTGTCATGACGTTGACC-3’ | LC127055 | Present study |
| *goosecoid* | 5’-ACCCCGCAGCACATCCCGGCCTACTA-3’  5’-GACGAGACGCTACGGACGTCATCCTC-3’ | LC131329 | Present study |
| *lefty* | 5’-GCAGCAACATCGCATCTTAC-3’  5’-ACGCACACTGTTCTACGATC-3’ | LC127056 | Present study |
| *nodal* | --- | AB097411 | Direct submission (Saiga et al., 2003) |
| *not-like* | --- | LC127057 | From Dr. Ichiro Masai |
| *wnt8* | --- | AF206500 | Yasui et al. (2001)[34] |

K, G/T; R, A/G; S, C/G.
