# Supplementary material for: Acquisition of the dorsal structures in chordate amphioxus
Source: Open Biol. 2016 Jun 15;6(6):160062. doi: 10.1098/rsob.160062 (PMC4929940; doi:10.1098/rsob.160062)
Supplement: Figures S1, S2, S3, S4, and S5 [file rsob160062supp2.pdf]

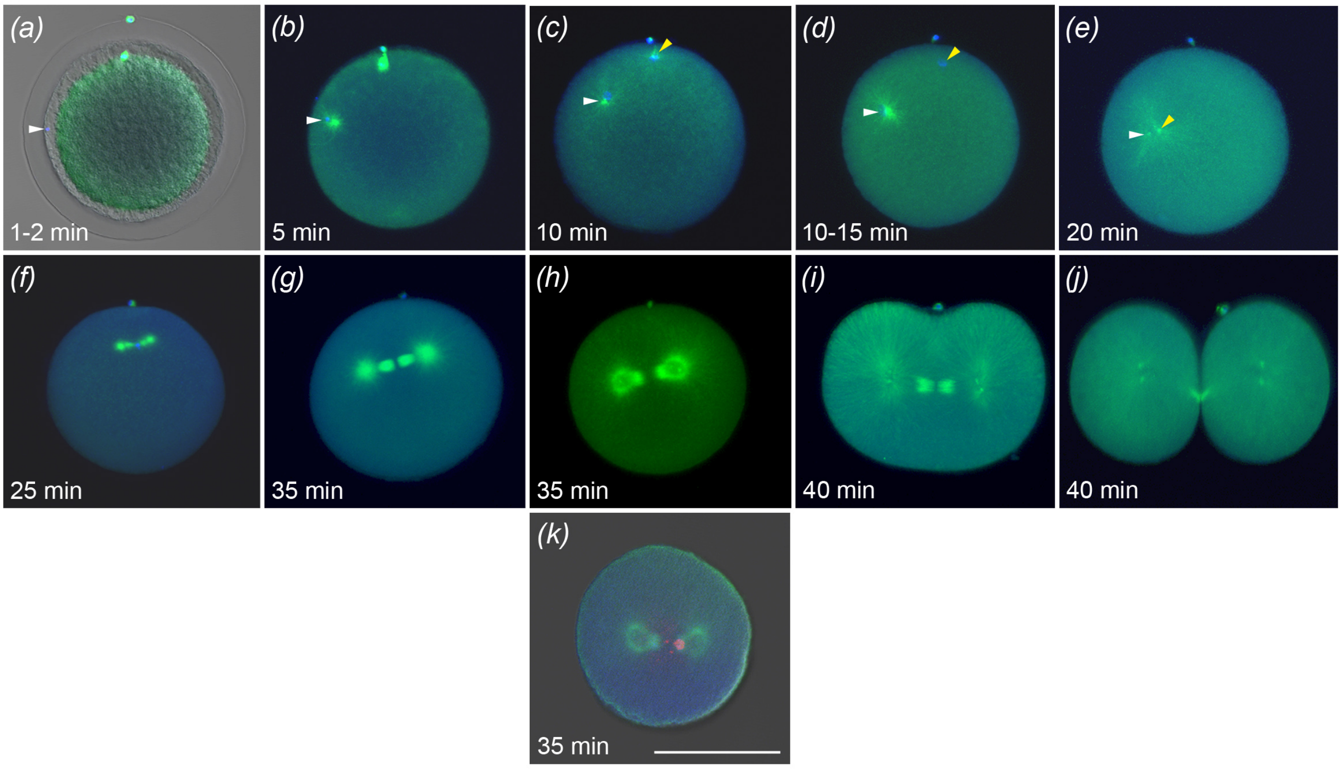

Figure S1. Pronuclear migration and first cleavage. DNA and microtubules were labelled with Hoechst (blue) and Alexa Fluor 488 (green). (*a*-*e*) Pronuclear migration. White and yellow arrowheads denote male and female pronucleus, respectively. (*f*-*k*) First cleavage. All but (*k*) are lateral view setting animal pole (polar body) to the top. (*k*) Animal view with actin filaments in polar body labeled with Alexa Fluor 594 (red). Male and female pronuclei meet on one side in animal hemisphere just above equator around 20 mpf (*e*). Chromosomes are aligned at the vicinity of the centre with some angle to equatorial plane (*h*, *k*).

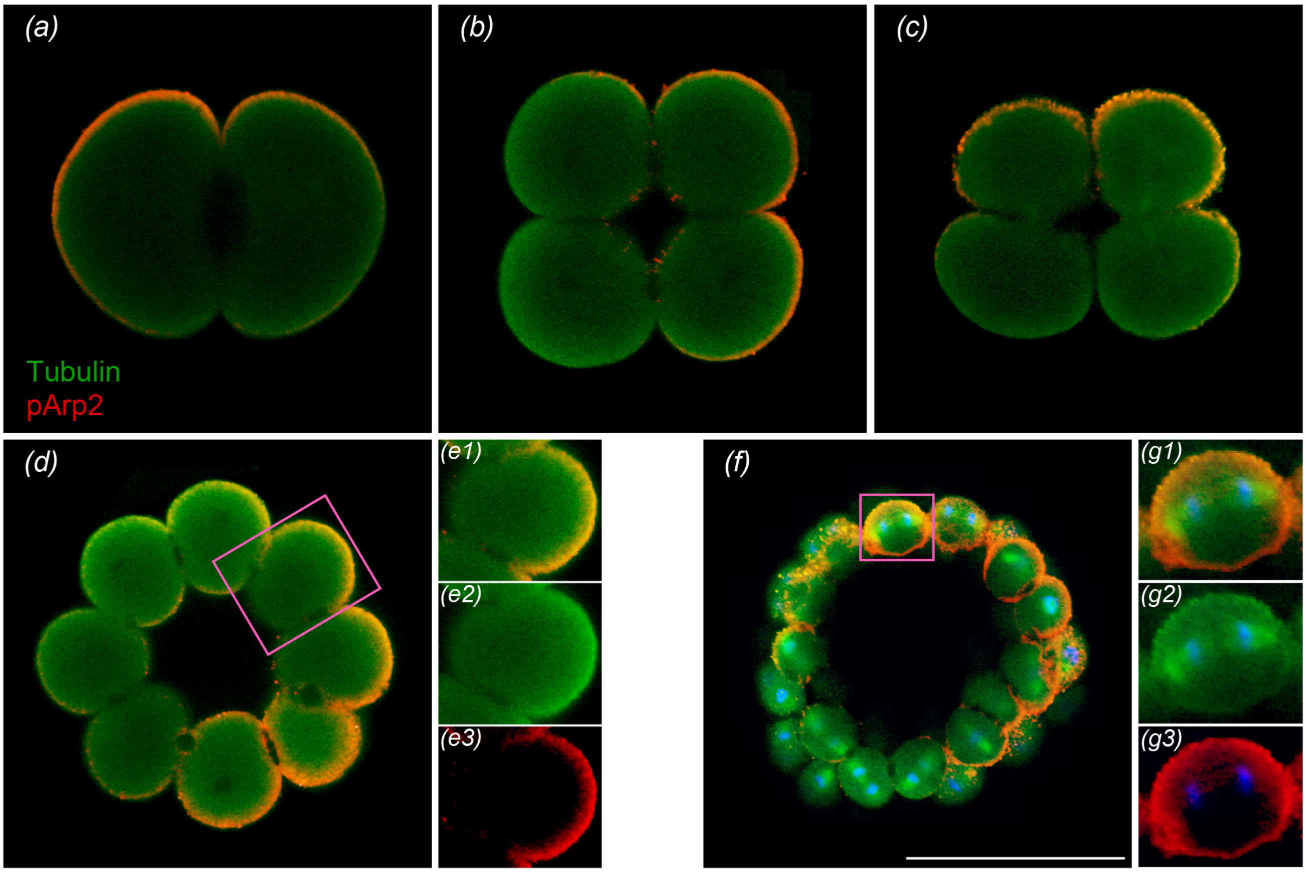

Figure S2. Co-localization of cortical microtubules and active Arp2/3 complex at cleavage stages. Partial renderling images at 2-cell (*a*), 4-cell (*b*), 8-cell (*c*), 16-cell (*d*), and 32-64-cell stage. (*e1-3*) and (*g1-3*) are magnifications of merged, anti-α-tubulin and anti-pArp2 immunopositive signal images indicated by rectangles in (*d*) and (*f*), respectively. Blue in (*f*, *g1-3*) is DNA stained with Hoecst. Scale bar 100 μm.

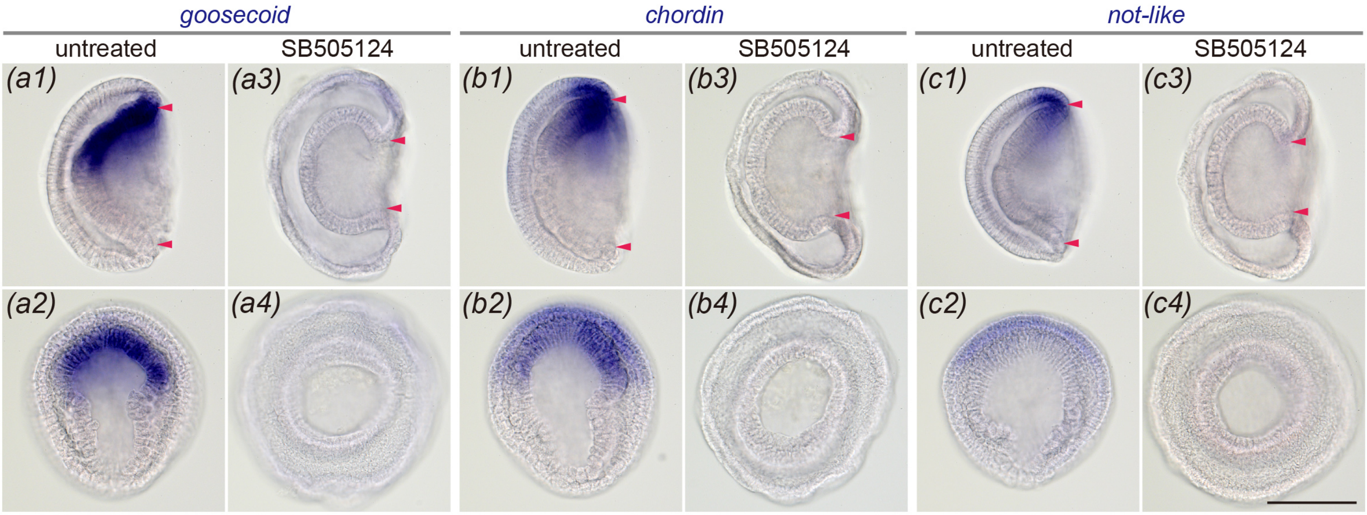

Figure S3. Expression patterns of Nodal signalling downstream genes. Dorsal to the top. (*a1, a2*, *b1, b2*, *c1, c2*) Untreated mid-gastrulae. (*a3, a4*, *b3, b4*, *c3, c4*) SB505124-treated mid-gastrulae. The treatment was performed at 50 μM concentration soon after fertilization to mid-gastrula stage. Arrowheads denote shrunken blastopore. Lateral view in upper array and blastopore view in lower array. Scale bar 100 μm.

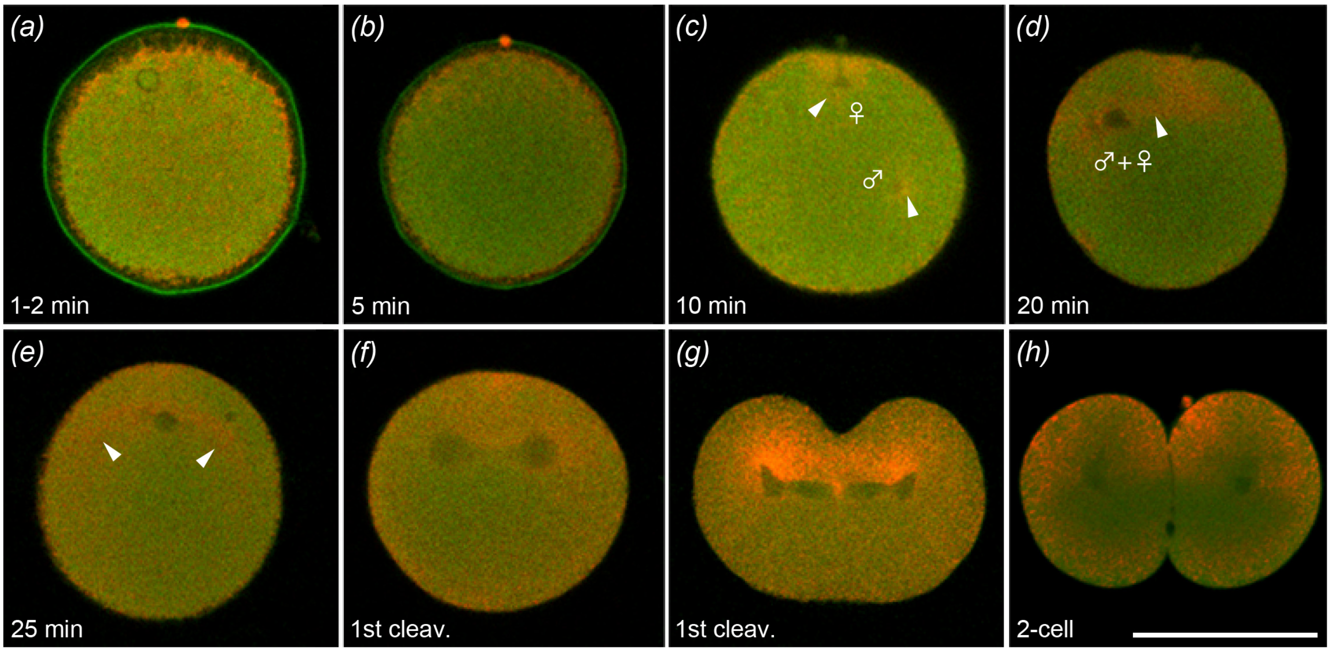

Figure S4. Mitochondrial distribution during pronuclear migration, syngamy, and first cleavage. Mitochondria and ER are labelled with MitoTracker (orange) and ER Tracker (green), respectively. Rendering images scanned about a half of cell diameter. Animal pole to the top. (a) Almost uniformly scattered dot distribution in cortical region at sperm fusion. (b) Animal distribution at 5 mpf. (c) Accumulation around male and female pronuclei (arrowheads) at 10 mpf. (d) Initiation of arch-shaped distribution through fusing pronuclei (arrowhead) at 20 mpf. (e) Arch-shaped distribution at 25 mpf (arrowheads). (f-h) Animal distribution above nuclei during first cleavage. Scale bar 100 µm.

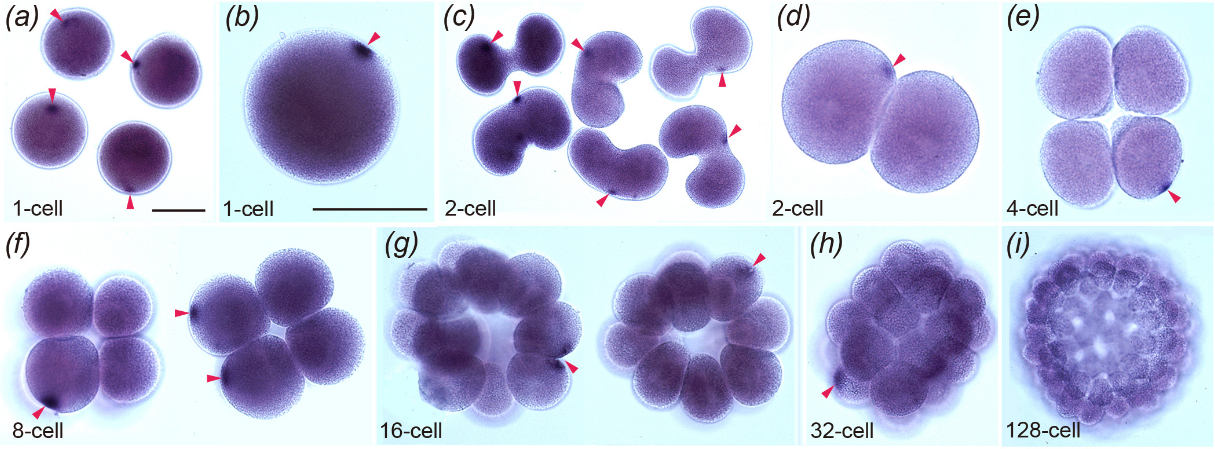

Figure S5. Spotted distribution of maternal *tbrain* (*eomesodermin*) mRNA. Expression pattern was detected by WISH. (*a*, *b*) Strong signal spot with ubiquitous weak expression in fertilized eggs. (*c*) Signal spot is located near vegetal pole in cleaving embryos. (*d*-*h*) Signal spot is located in a blastomere but rarely in two blastomeres in 4- to 32-cell embryos. (*i*) Signal spot is not observed at 128-cell stage. Arrowheads denote signal spots. RNA probe was synthesised with cDNA (BAB63370, Satoh et al., 2002). Scale bar 100 μm.
